# IL-1β mediates MCP-1 induction by Wnt5a in gastric cancer cells

**DOI:** 10.1186/1471-2407-14-480

**Published:** 2014-07-03

**Authors:** Shengjun Li, Wei Wang, Ning Zhang, Tingxian Ma, Chenghai Zhao

**Affiliations:** 1 Department of Immunology, College of Basic Medical Science, China Medical University, Shenyang, China; 2 Department of Pathophysiology, College of Basic Medical Science, China Medical University, Shenyang, China

## Abstract

**Background:**

Both Wnt5a overexpression and macrophage infiltration have been implicated in inflammation and cancer. The aim of this study is to reveal the involvement of Wnt5a in macrophage recruitment in gastric cancer.

**Methods:**

mRNA expression in gastric cancer tissues and cells was investigated by real-time PCR. Protein secretion by gastric cancer cells was determined by ELISA. PcDNA3.1-Wnt5a expression vector and Wnt5a siRNA vector were used to overexpress and silence Wnt5a expression in gastric cells, respectively. Macrophage migration was analyzed by transwell, and macrophage cytoskeleton was stained with FITC-phalloidin.

**Results:**

Wnt5a was overexpressed in gastric cancer tissues, and correlated with monocyte chemotactic protein 1 (MCP-1) and interleukin 1β (IL-1β), respectively. In gastric cancer cells, Wnt5a induced MCP-1 expression, which was mediated by IL-1β. Conditioned medium from gastric cancer cells transfected with Wnt5a stimulated macrophage chemotaxis and cytoskeletal changes via MCP-1, which were suppressed by recombinant IL-1 receptor antagonist (rIL-1Ra).

**Conclusions:**

These results suggest that Wnt5a is involved in macrophage recruitment by upregulating MCP-1, and IL-1Ra may be used to inhibit macrophage recruitment in gastric cancer.

## Background

Roles of noncanonical Wnt5a in human malignancies seem to mainly depend on its action on canonical Wnt/β-catenin pathway. In some conditions, Wnt5a functions as a tumor suppressor by antagonizing the oncogenic β-catenin dependent pathway [1-3]. However, in other conditions, Wnt5a has no effect on β-catenin signaling, and acts as a tumor promoter due to its stimulatory action on tumor migration and invasion [4,5]. Actually, Wnt5a may play more complicated roles in human tumors. Recent studies have demonstrated that Wnt5a is implicated in inflammation. Some inflammatory stimuli were found to trigger the production of Wnt5a, which subsequently induced the secretion of inflammatory cytokines [6-8]. Acute inflammatory response is favorable for the eradication of pathogens and tumor cells, whereas chronic inflammation with persistent existence of inflammatory cytokines is closely linked to carcinogenesis [9,10].

Macrophage infiltration is frequently observed in tumor tissues, which plays a dual role in tumor initiation and progression. Activated macrophages can directly remove tumor cells, or destroy tumor cells via secreted mediators, such as cytokines and nitric oxide. However, long-standing macrophages may undergo phenotype changes, and produce tumor-promoting cytokines, chemokines and growth factors [11]. Macrophage recruitment in tumor microenvironment is usually attributed to some chemokines which are produced by both tumor cells and stromal cells. In gastric inflammation and cancer, macrophage infiltration is associated with the overproduction of monocyte chemotactic protein 1 (MCP-1) [12,13].

Induction of MCP-1 is related to some inflammatory cytokines, such as interleukin 1β (IL-1β) and tumor necrosis factor α (TNF-α) [14-17]. These cytokines are overproduced in gastric inflammation and cancer,however, it remains unknown whether these cytokines regulate MCP-1 expression in gastric epithelial cells. Furthermore, it is not clear whether Wnt5a stimulates the production of these cytokines. In the present study, we investigated the effect of Wnt5a on MCP-1 production and macrophage chemotaxis to evaluate the involvement of Wnt5a in macrophage recruitment in gastric epithelium.

## Methods

### Gastric cancer cell lines and tissue specimens

Human gastric cancer cells BGC-803, HGC-27 and MKN-45 were cultured in RPMI 1640 supplemented with 10% fetal bovine serum, at 37°C in a humid incubator with 5% CO_2_. 50 primary gastric cancer specimens with matched adjacent non-malignant tissues were acquired from patients under operation with all their informed consent at Shengjing hospital, Chinese Medical University. Haematoxylin- and eosin-staining sections were prepared for assessment of the percentage of tumor cells. 36 specimens with >80% tumor cells were selected for analysis, in which 19 were *Helicobacter pylori* (*H. pylori*) positive. This study was carried out with the approval of the ethical committee of China Medical University. All experiments were done at least three times.

### Macrophage cell line RAW264.7

Macrophage RAW 264.7 was obtained from the American Type Culture Collection (Rockville, MD, USA), and was cultured in Dulbecco’s modified Eagle’s medium, supplemented with 10% fetal bovine serum, at 37°C in a humid incubator with 5% CO_2_.

### Real-time PCR

RNA was isolated from cells using TRIzol® LS Reagent (Invitrogen Life Technologies) according to the manufacturer’s protocol. 1 μg RNA was reverse transcribed into cDNA using Superscript III reverse transcriptase (Invitrogen Life Technologies). Real-time PCR was carried out in the LightCycler system (Roche Diagnostics) with LightCycler DNA Master SYBR Green I Kit (Roche Diagnostics). Gene expression was quantified by the comparative CT method, normalizing CT values to GAPDH which was used as an internal control. Primer sequences for Wnt5a were described in [8], for MCP-1 in [13], and for IL-1β, interleukin 6 (IL-6), TNF-α and glyceraldehyde-3-phosphate dehydrogenase (GAPDH) in [18].

### ELISA

Concentrations of MCP-1 and cytokines in cell (2 × 10^5^) culture supernatants were analyzed with Quantikine ELISA kits (Boster, Wuhan, China) according to the manufacturer’s instructions.

### Expression vector transfection

The pcDNA3.1 (Invitrogen, Paisley, United Kingdom) Wnt5a expression vector was made by cloning of the full-length PCR product of Wnt5a with PFU DNA polymerase (Invitrogen, Paisley, United Kingdom). Cells were transfected with 2 μg pcDNA3.1-Wnt5a expression vector or 2 μg pcDNA3.1 empty vector per well at 70% confluence using Lipofectamine 2000 reagent (Invitrogen, Paisley, United Kingdom) according to the manufacturer’s protocol.

### RNA interference

Wnt5a siRNA vector and nonsilencing control siRNA vector were acquired from Takala (Dalian, China). Cells were seeded into a 24-well plate at a density of 2 × 10^5^. On the following day, cells were transfected with siRNA vector using Lipofectamine 2000 (Invitrogen, United Kingdom) according to the manufacturer’s instructions.

### Macrophage chemotaxis assay

Transwell (COSTAR, 24-well, 8 μm pore) was used to analyze macrophage chemotaxis. Macrophages (2 × 10^5^) were added to the upper chamber with the addition of conditioned medium into the lower chamber, and then incubated for 8 hours at 37°C and 5% CO_2_. Migrated macrophages were fixed with 4% paraformaldehyde and stained with crystal violet according to the manufacturer’s protocol. The number of migrated macrophages in five random microscopy fields was counted.

### Cytoskeletal staining

Macrophages (2 × 10^5^) were seeded on 24-well culture plates, washed with PBS and fixed with 4% paraformaldehyde for 20 minutes. Then macrophages were incubated with 0.2% Triton X-100 for 10 minutes. After blocking with 1% bovine serum albumin, cells were incubated with FITC-phalloidin (Sigma) for 40 minutes. Images were captured by confocal fluorescent microscopy.

### Statistical analysis

Mann-Whitney *U*-test was used to compare mRNA expression between gastric cancer tissues and adjacent non-malignant tissues, and between *H.pylori*-positive and *H. pylori*-negative cancer tissues. Correlation among Wnt5a, MCP-1 and IL-1β in gastric cancer tissues was analyzed using Spearman’s rank correlation test. Differences among cells were analyzed by Student’s *t*-test or one way ANOVA. SPSS version 11.0 (SPSS, Chicago, IL, USA) was used to perform statistical analysis. A *P*-value less than 0.05 was considered significant.

## Results

### Wnt5a mRNA was overexpressed in gastric cancer tissues

**Figure 1:**
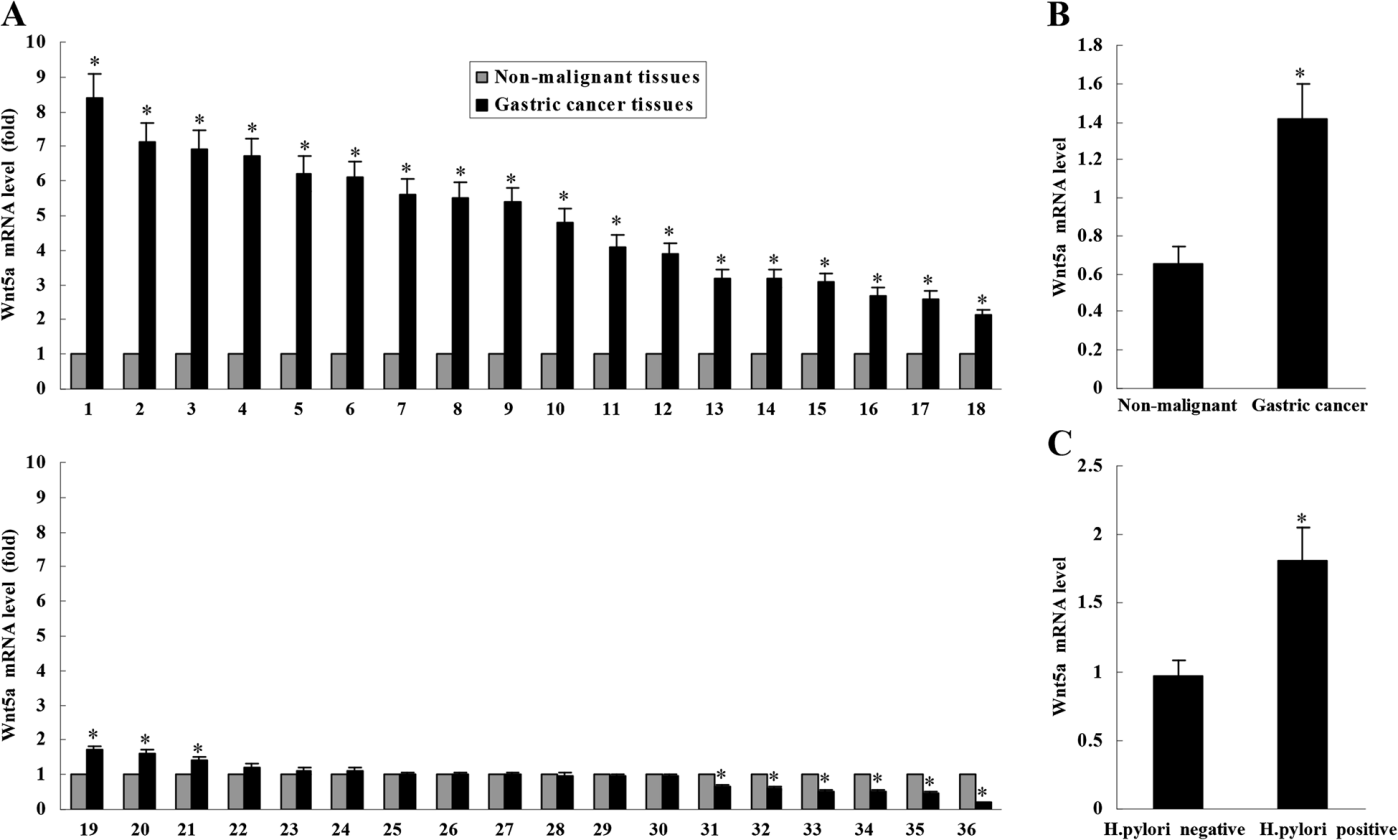
**Wnt5a expression in gastric cancer tissues. (A)** Wnt5a mRNA expression was upregulated in 21 gastric cancer specimens, whereas downregulated in 6 cases by real-time PCR, compared with matched non-malignant tissues. *P < 0.01 *vs* Non-malignant tissues. **(B)** Wnt5a mRNA level in cancer tissues was significantly higher than that in adjacent non-malignant tissues. *P < 0.01 *vs* Non-malignant. **(C)** Wnt5a mRNA level in *H. pylori*-positive cancer tissues was higher than that in *H. pylori*-negative ones. *P < 0.05 *vs H. pylori* negative.

Real-time PCR was used to determine Wnt5a mRNA level in 36 gastric cancer specimens and matched adjacent non-malignant tissues. Wnt5a expression in each specimen was standardized to GAPDH expression. Compared with matched non-malignant tissues, Wnt5a mRNA was upregulated in 21 gastric cancer specimens, whereas downregulated in 6 cases (Figure 1A). Wnt5a mRNA level in cancer tissues was significantly higher than that in adjacent non-malignant tissues (Figure 1B). Moreover, Wnt5a mRNA level in *H. pylori*-positive cancer tissues was higher than that in *H. pylori*-negative ones (Figure 1C).

### Wnt5a stimulated MCP-1 expression in gastric cancer cells

**Figure 2:**
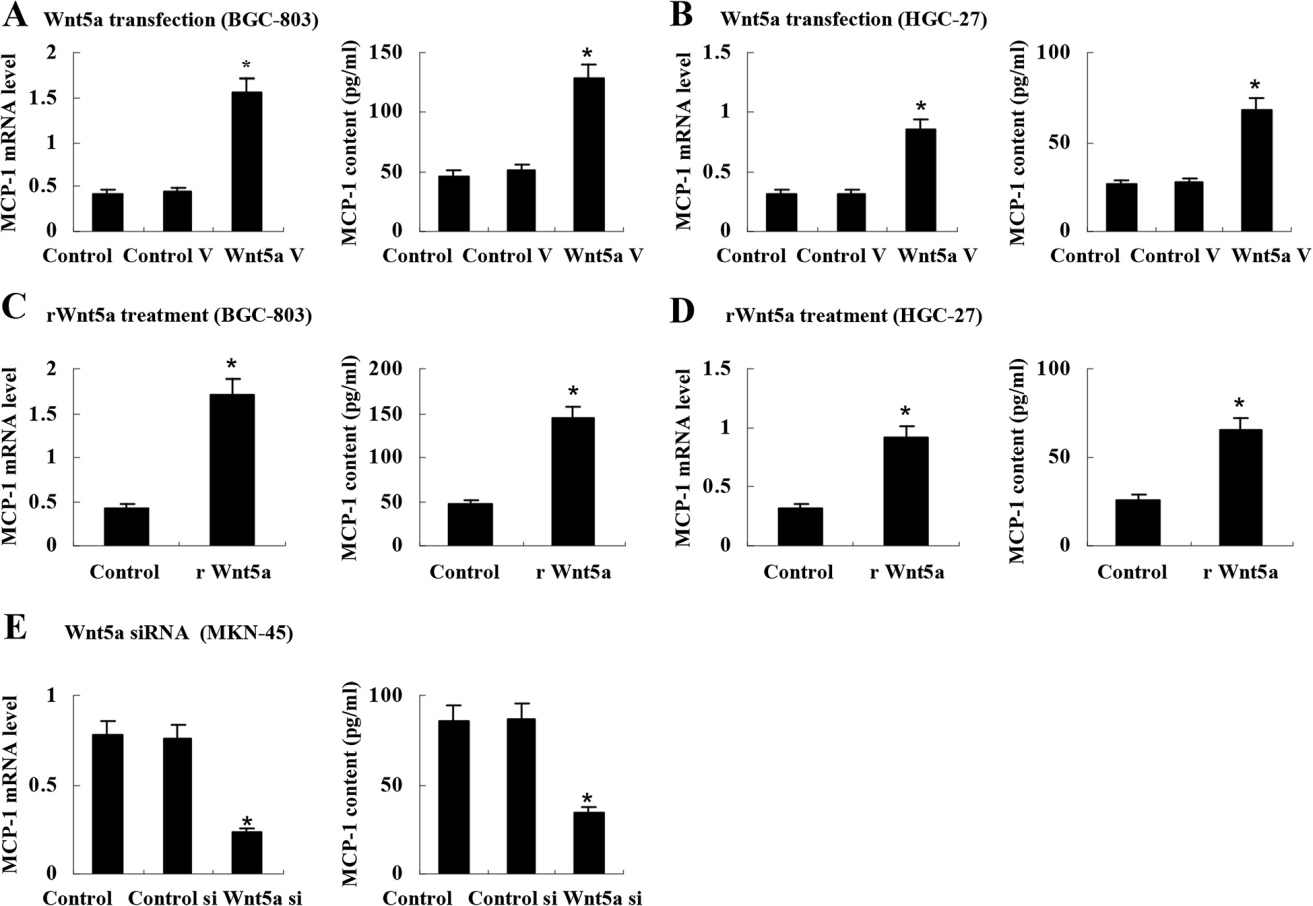
**Stimulation of MCP-1 expression by Wnt5a in gastric cancer cells. (A)** and **(B)** Wnt5a transfection induced MCP-1 expression in BGC-803 cells and HGC-27 cells by real-time PCR and ELISA. **(C)** and **(D)** Recombinant Wnt5a (0.5 μg/ml) treatment promoted MCP-1 expression in BGC-803 cells and HGC-27 cells. **(E)** Wnt5a siRNA suppressed MCP-1 expression in MKN-45 cells. *P < 0.01 *vs* Control. Control V: control vector; Wnt5a V: Wnt5a vector; rWnt5a: recombinant Wnt5a. Control si: control siRNA; Wnt5a si: Wnt5a siRNA. Data are expressed as mean ± SD, n = 3.

To explore the role of Wnt5a overexpression in macrophage recruitment, we investigated the effect of Wnt5a on the expression of MCP-1, a chemoattractant for macrophages, in gastric cancer cell lines. After transfection with Wnt5a expression vector (pcDNA3.1-Wnt5a) for 48 hours, BGC-803 cells expressed more MCP-1 mRNA and secreted more MCP-1 protein into cell supernatant (Figure 2A). To confirm the stimulatory effect of Wnt5a on MCP-1 expression, we next transfected another gastric cancer cell HGC-27 with pcDNA3.1-Wnt5a. Similar to BGC-803 cells, Wnt5a-transfected HGC-27 cells overexpressed MCP-1 (Figure 2B). Furthermore, we treated BGC-803 and HGC-27 cells with recombinant Wnt5a (rWnt5a, 0.5 μg/ml, R&D system) for 8 hours. Consistent with Wnt5a transfection, rWnt5a treatment also increased MCP-1 expression (Figure 2C and 2D). On the other side, we silenced Wnt5a expression in Wnt5a-positive gastric cancer cell line MKN-45 [8] with Wnt5a siRNA vector. As expected, MCP-1 expression was downregulated (Figure 2E).

### Wnt5a induced IL-1β expression in gastric cancer cells

**Figure 3:**
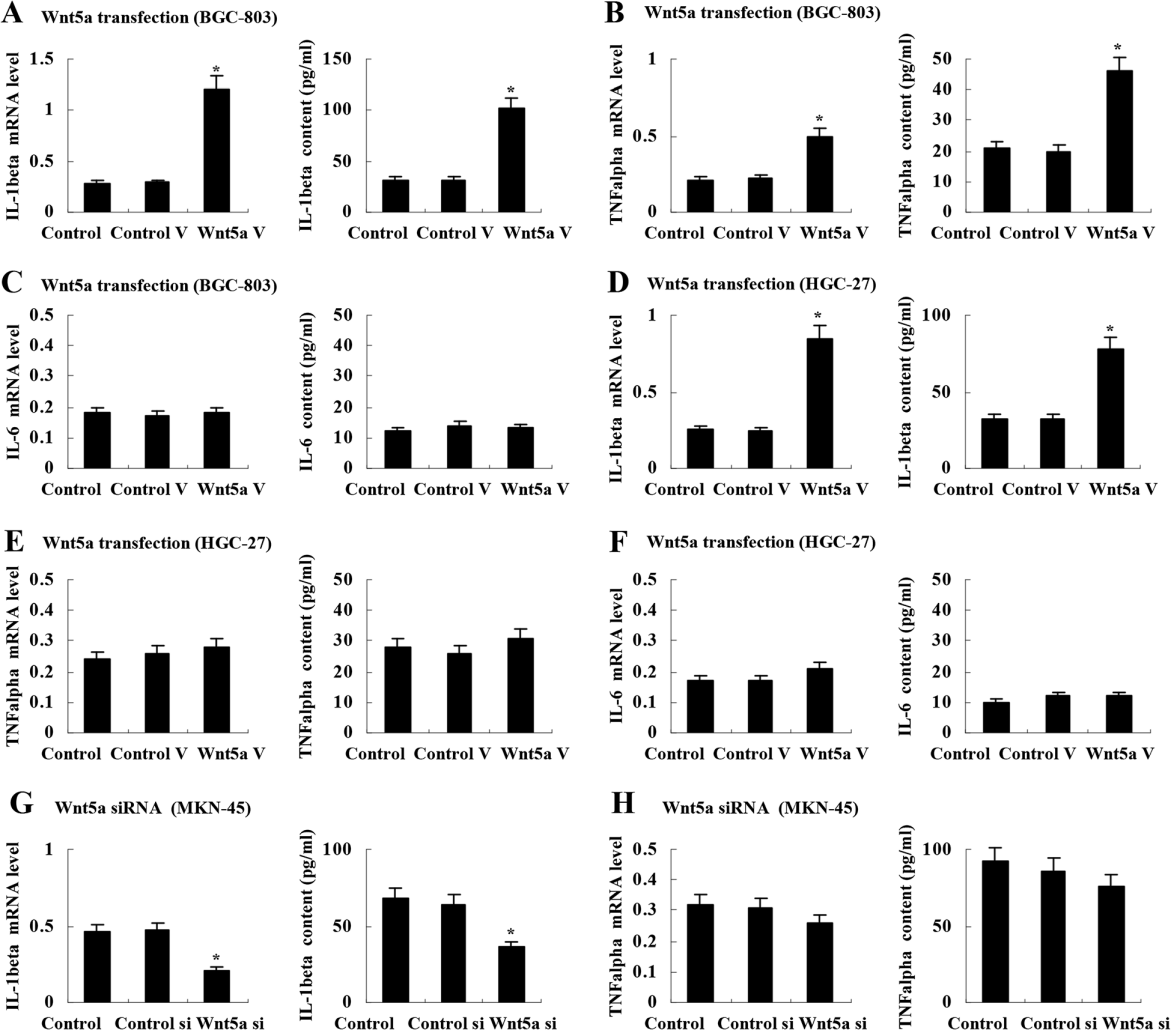
**Induction of IL-1β expression by Wnt5a in gastric cancer cells. (A)** and **(B)** Wnt5a transfection induced IL-1β and TNF-α expression in BGC-803 cells by real-time PCR and ELISA. **(C)** Wnt5a transfection had no effect on IL-6 expression in BGC-803 cells. **(D)** Wnt5a transfection stimulated IL-1β expression in HGC-27 cells. **(E)** and **(F)** Wnt5a transfection had no effect on TNF-α and IL-6 expression in HGC-27cells. **(G)** Wnt5a siRNA inhibited IL-1β expression in MKN-45 cells. **(H)** Wnt5a siRNA has no effect on TNF-α expression in MKN-45 cells. *P < 0.01 *vs* Control. Control V: control vector; Wnt5a V: Wnt5a vector. Control si: control siRNA; Wnt5a si: Wnt5a siRNA. Data are expressed as mean ± SD, n = 3.

To elucidate the mechanisms underlying MCP-1 induction by Wnt5a, we investigated the expression of some inflammatory cytokines which have been shown to be involved in MCP-1 upregulation. In BGC-803 cells, Wnt5a transfection led to a significant increase in IL-1β and TNF-α expression (Figure 3A, 3B), but not in IL-6 (Figure 3C). In addition, IL-1β expression was also upregulated in Wnt5a-transfected HGC-27 cells (Figure 3D-3F). To the contrary, IL-1β expression was downregulated in MKN-45 cells treated with Wnt5a siRNA vector (Figure 3G and 3H). Some other cytokines were further examined, including interleukin 4 (IL-4), interleukin 8 (IL-8), interleukin 10 (IL-10), Prostaglandin E_2_ (PGE_2_) and interferon γ (IFN-γ). ELISA detection showed that Wnt5a had no effect on the expression of these cytokines in gastric cancer cells.

### IL-1β mediated Wnt5a-induced MCP-1 upregulation in gastric cancer cells

**Figure 4:**
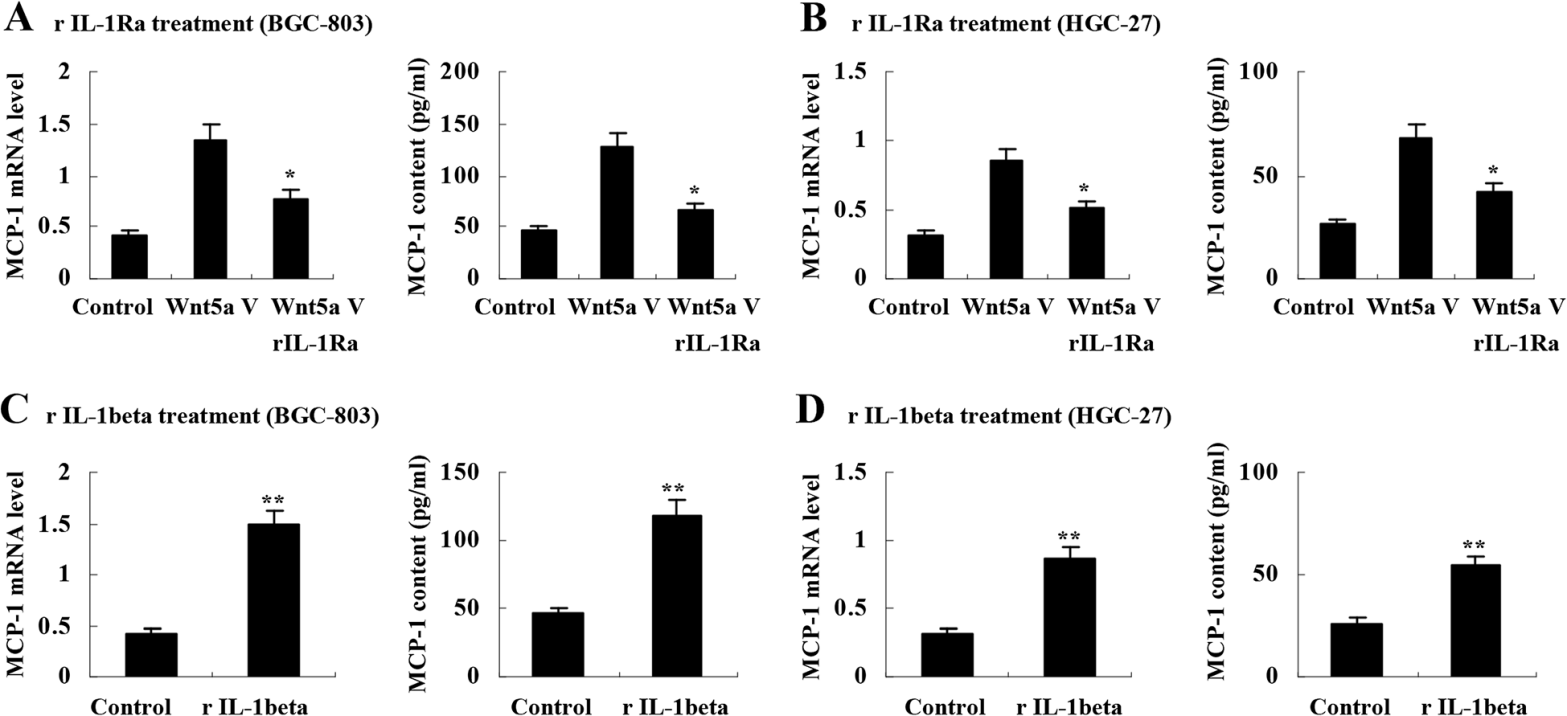
**Mediation of IL-1β in Wnt5a-induced MCP-1 upregulation in gastric cancer cells. (A)** and **(B)** rIL-1Ra (0.5 μg/ml) inhibited MCP-1 upregulation by Wnt5a transfection in BGC-803 cells and HGC-27 cells. **(C)** and **(D)** rIL-1β (1 μg/ml) stimulated MCP-1 expression in BGC-803 cells and HGC-27 cells. *P < 0.01 *vs* Wnt5a V; **P < 0.01 *vs* Control. Control V: control vector; Wnt5a V: Wnt5a vector; rIL-1Ra: recombinant IL-1 receptor antagonist; rIL-1beta: recombinant IL-1β. Data are expressed as mean ± SD, n = 3.

To determine whether IL-1β was involved in MCP-1 upregulation by Wnt5a, we treated Wnt5a-transfected BGC-803 cells with recombinant IL-1 receptor antagonist (rIL-1Ra, 0.5 μg/ml, Sigma) for 8 hours. It was found that MCP-1 upregulation by Wnt5a transfection was significantly inhibited (Figure 4A). Wnt5a-transfected HGC-27 cells were also treated with rIL-1Ra (0.5 μg/ml) to verify the role of IL-1β in MCP-1 upregulation. As expected, Wnt5a-induced MCP-1 overexpression in HGC-27 cells was suppressed as well (Figure 4B). In addition, we used recombinant IL-1β (rIL-1β, 1 μg/ml, Sigma) to treat BGC-803 cells and HGC-27 cells, and found that MCP-1 expression was upregulated both in mRNA transcription and protein secretion in these cells (Figure 4C and 4D).

### Wnt5a-conditioned medium promoted macrophage chemotaxis

**Figure 5:**
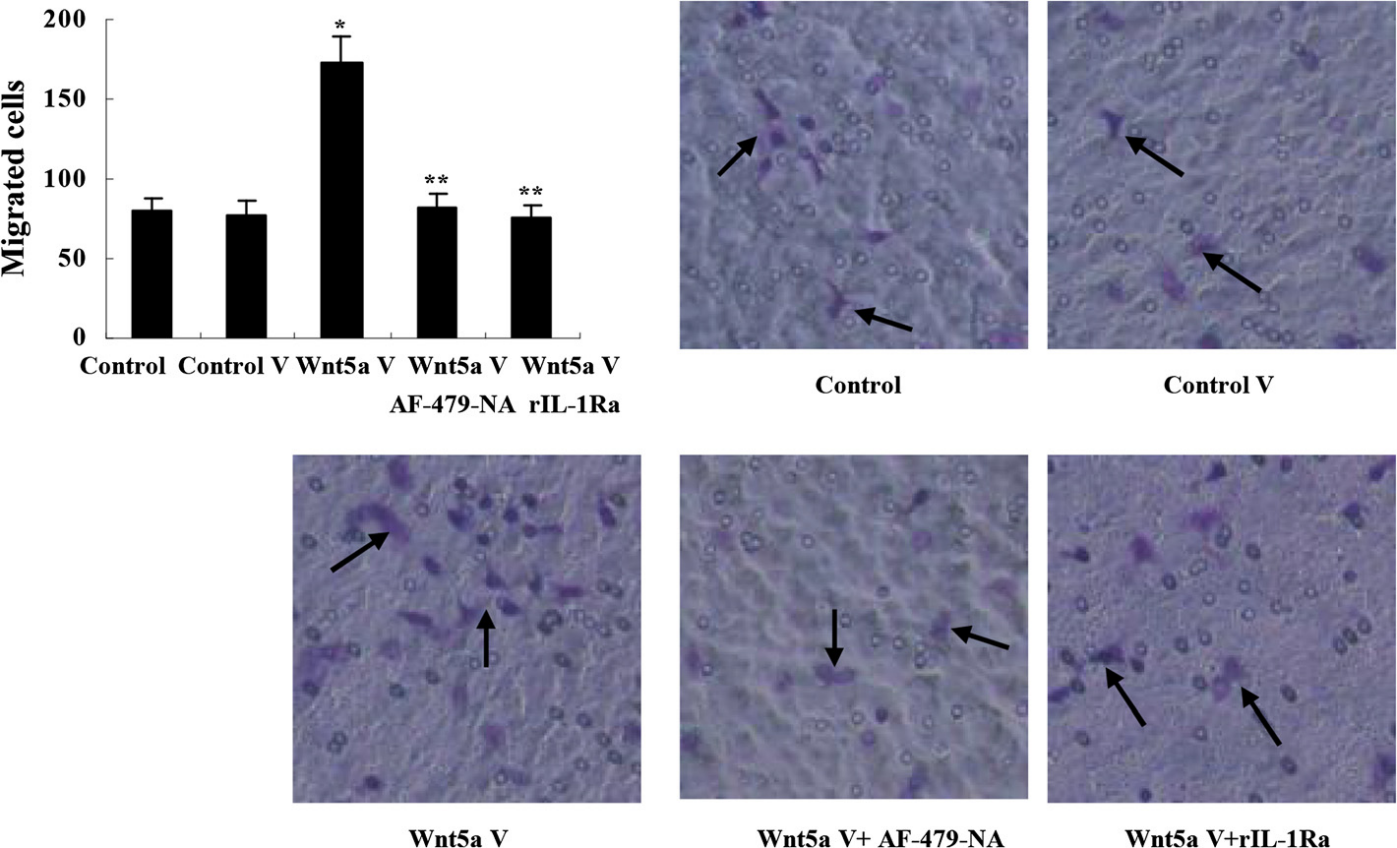
**Promotion of macrophage chemotaxis by Wnt5a-conditioned medium.** Wnt5a-conditioned medium from Wnt5a-transfected BGC-803 cells enhanced macrophage migration by transwell analysis, which was inhibited by the addition of MCP-1 neutralizing antibody AF-479-NA (0.1 μg/ml) into the medium or the pretreatment of Wnt5a-transfected BGC-803 cells with rIL-1Ra. *P < 0.01 *vs* Control; **P < 0.01 *vs* Wnt5a V. Control V: control vector; Wnt5a V: Wnt5a vector; rIL-1Ra: recombinant IL-1 receptor antagonist. Data are expressed as mean ± SD, n = 3.

To evaluate whether the increased MCP-1 secretion was functional, macrophage migration was assayed in vitro. It was observed that the number of migrated macrophages from transwell upper chamber increased significantly when Wnt5a-conditioned medium from BGC-803 cells transfected with Wnt5a expression vector was added to the lower chamber (Figure 5). To clarify the role of MCP-1 in the increased cell migration, we added MCP-1 neutralizing antibody AF-479-NA (0.1 μg/ml, R&D Systems) into Wnt5a-conditioned medium. It was found that the increased cell migration was inhibited remarkably (Figure 5). Moreover, the enhanced macrophage migration by Wnt5a-conditioned medium was suppressed when Wnt5a-transfected BGC-803 cells were pretreated with rIL-1Ra (Figure 5).

### Wnt5a-conditioned medium induced macrophage cytoskeletal changes

**Figure 6:**
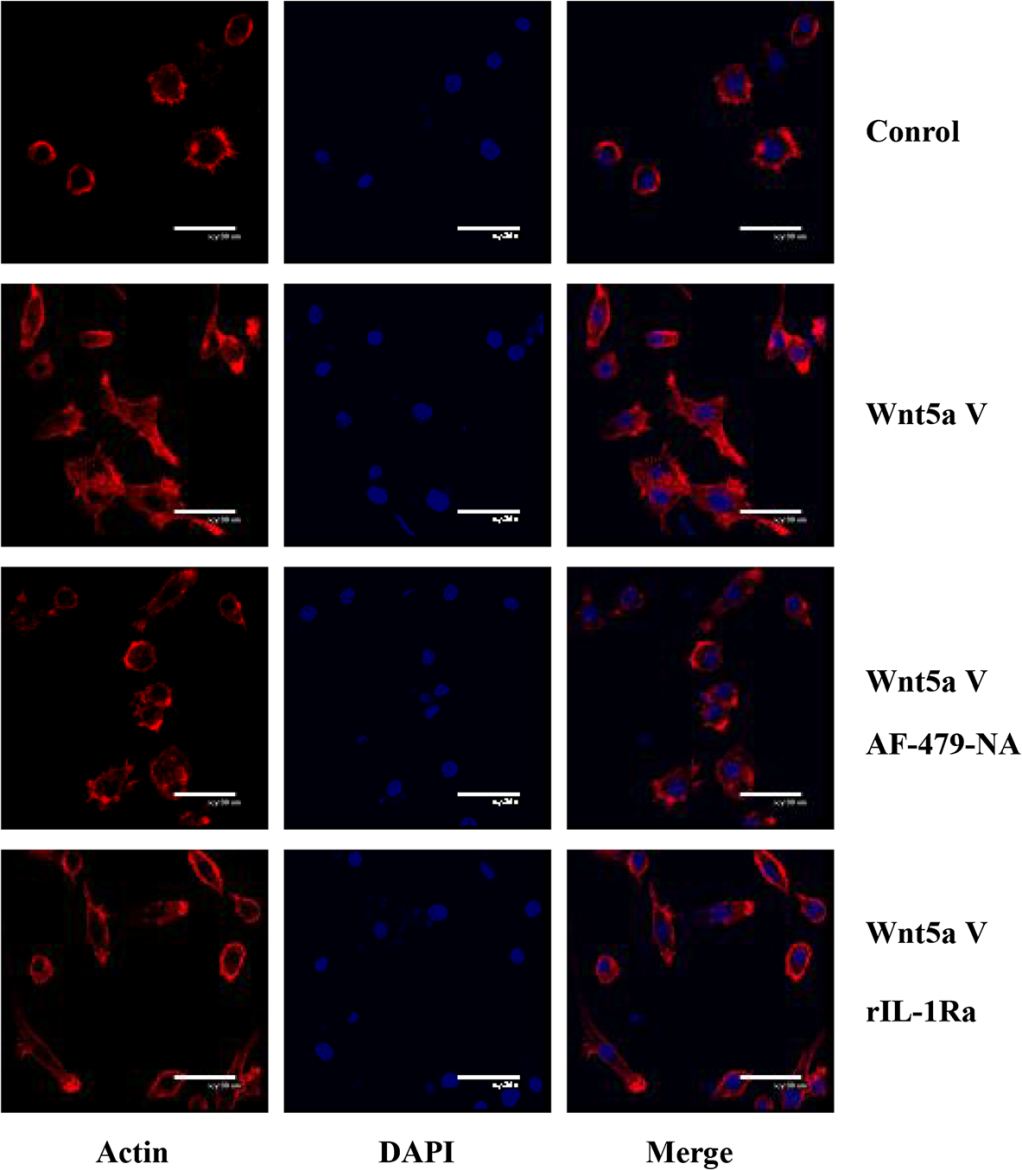
**Induction of macrophage cytoskeletal changes by Wnt5a-conditioned medium.** Wnt5a-conditioned medium from Wnt5a-transfected BGC-803 cells induced macrophage cytoskeletal changes by FITC-phalloidin and DAPI staining, which was inhibited by the addition of MCP-1 neutralizing antibody AF-479-NA (0.1 μg/ml) into the medium or the pretreatment of Wnt5a-transfected BGC-803 cells with rIL-1Ra. Wnt5a V: Wnt5a vector; rIL-1Ra: recombinant IL-1 receptor antagonist.

We next assessed the effect of Wnt5a-conditioned medium from BGC-803 cells on macrophage cytoskeleton. The cytoskeleton was stained using FITC-phalloidin (Sigma). Consistent with migration assays, Wnt5a-conditioned medium induced significant cytoskeletal changes, compared with control medium (Figure 6). However, cytoskeletal changes were inhibited by MCP-1 neutralizing antibody AF-479-NA (0.1 μg/ml) (Figure 6). Furthermore, when Wnt5a-transfected BGC-803 cells were pretreated with rIL-1Ra, cytoskeletal changes induced by the conditioned medium were also suppressed (Figure 6).

### Correlation among Wnt5a, MCP-1 and IL-1β in gastric cancer tissues

**Figure 7:**
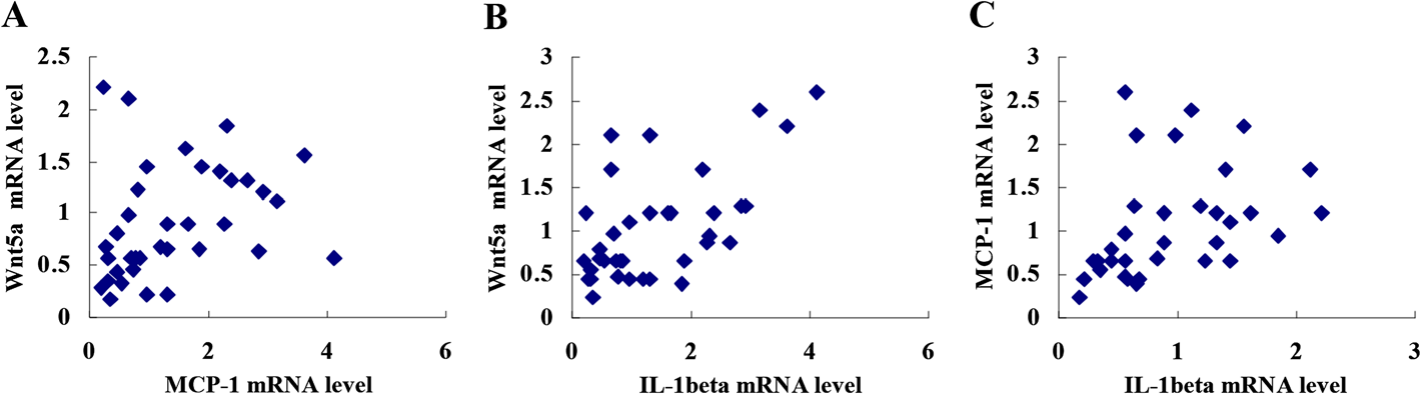
**Correlation among Wnt5a, MCP-1 and IL-1β in gastric cancer tissues. (A)** Wnt5a mRNA expression was correlated with MCP-1 mRNA expression in gastric cancer tissues by Spearman’s rank correlation test. *P* = 0.019. **(B)** Wnt5a mRNA expression was correlated with IL-1β mRNA expression. *P* = 0.039. **(C)** MCP-1 mRNA expression was correlated with IL-1β mRNA expression, *P* = 0.011.

Spearman’s rank correlation test was used to analyze the correlation among Wnt5a, MCP-1 and IL-1β in gastric cancer tissues. It was found that Wnt5a mRNA expression was correlated with MCP-1 mRNA expression (Figure 7A) and IL-1β mRNA expression (Figure 7B), respectively. Moreover, MCP-1 mRNA expression was correlated with IL-1β mRNA expression (Figure 7C).

## Discussion

This study reveals that Wnt5a is overexpressed in gastric cancer tissues in mRNA level, which is consistent with another study showing Wnt5a overexpression in protein level by immunohistochemistry [19], however, Wnt5a overexpression has not been found in some gastric cancer cell lines [20]. This discrepancy may be due to the cancer-stromal interaction in gastric cancer microenvironment. Actually, TNF-α was shown to induce Wnt5a [21], and a nuclear factor-kappa B (NF-κB) binding site was found in Wnt5a promoter [22], suggesting *H. pylori* as an inducer of Wnt5a in gastric epithelium. Recently, Wnt5a has been shown to be induced by inflammatory stimuli in other cells, such as macrophages [23,24] and bone marrow mesenchymal cells (BMSC) [6].

Increasing evidence indicates Wnt5a as a pro-inflammatory mediator due to its involvement in some inflammatory processes and diseases, and its stimulatory action on inflammatory cytokine production. Wnt5a was upregulated in the Alzheimer’s disease mouse brain due to β-amyloid peptide, inducing neuroinflammation and neurotoxicity via inflammatory cytokines IL-1β and TNF-α [25]. Wnt5a was also involved in Human Immunodeficiency Virus (HIV) associated neurological disorders by promoting IL-1β, IL-6 and TNF-α production in the spinal cord [26]. Moreover, Wnt5a overproduction in rheumatoid arthritis was related to the secretion of IL-1β and IL-6 in BMSC [6]. Consistent with the above studies, the present study shows that Wnt5a stimulates gastric epithelial cells to produce IL-1β, suggesting the involvement of Wnt5a in gastric inflammation.

Our results demonstrate that Wnt5a is an inducer of MCP-1 in gastric cancer cells. First, Wnt5a expression was correlated with MCP-1 expression in gastric cancer tissues. Second, Wnt5a overexpression and exogenous Wnt5a treatment elevated MCP-1 expression in gastric cancer cells. Third, Wnt5a knockdown suppressed MCP-1 expression. Moreover, Wnt5a-conditioned medium stimulated macrophage chemotaxis and cytoskeletal changes, which was blocked by MCP-1 neutralizing antibody AF-479-NA. Taken together, our study suggests that Wnt5a may be involved in macrophage recruitment in gastric tissues via upregulating MCP-1.

The present study reveals a close link between MCP-1 and IL-1β in gastric cancer. MCP-1 expression was correlated with IL-1β expression in gastric cancer tissues. In gastric cancer cells, exogenous IL-1β stimulated MCP-1 expression, whereas IL-1Ra had an inhibitory effect. In addition, Wnt5a-induced macrophage chemotaxis and cytoskeletal changes were inhibited by IL-1Ra, suggesting IL-1β may mediate MCP-1 induction by Wnt5a. This observation is consistent with some other studies in which IL-1β stimulated MCP-1 expression in various types of cells, such as alveolar type II epithelial cells [14], peritoneal fibroblasts [15], endothelial cells [16] and preadipocytes [17].

The infiltrated macrophages seem to play complicated roles in the local tumor microenvironment upon interactions between cancer cells and macrophages. It is well known that these immunocytes can eradicate tumor cells, acting as tumor inhibitors. However, macrophages in tumor microenvironment may undergo alternative activation and change into tumor promoters called tumor associated macrophages (TAM). It has been demonstrated that tumor cells can “educated” macrophages to escape immune surveillance on one hand, and to create an environment fitful to growth on the other. Therefore, the role of Wnt5a in gastric cancer may be more complicated than we have known.

In summary, our study figures out a pathway that Wnt5a stimulates gastric epithelial cells to produce IL-1β in an autocrinal or paracrinal manner, and IL-1β induces gastric epithelial cells to secret MCP-1, which chemoattracts more macrophages into gastric mucosa. Moreover, this study has shown that rIL-1Ra can suppress Wnt5a-induced MCP-1 upregulation and macrophage chemotaxis, suggesting IL-1β blocking may be used to inhibit the aberrant macrophage infiltration, and suppress the development of chronic gastric inflammation and gastric cancer.

## Conclusions

Our results demonstrate that Wnt5a induces MCP-1 production in gastric cancer, which is mediated by IL-1β, suggesting that Wnt5a is involved in macrophage recruitment, and IL-1Ra may be used to inhibit macrophage recruitment in gastric cancer.

## Competing interests

The authors declare that they have no competing interests.

## Authors’ contributions

LS carried out PCR analysis,analyzed and interpreted the data, and drafted the manuscript. WW performed Elisa analysis. ZN performed Western analysis and expression vector transfection. MT performed macrophage migration analysis and macrophage cytoskeletal staining. ZC designed the study and drafted the manuscript. All authors read and approved the final manuscript.

## Pre-publication history

The pre-publication history for this paper can be accessed here:

http://www.biomedcentral.com/1471-2407/14/480/prepub
